# Mechanical Properties and Degradation Rate of Poly(Sorbitol Adipate-Co-Dioladipate) Copolymers Obtained with a Catalyst-Free Melt Polycondensation Method

**DOI:** 10.3390/polym16040499

**Published:** 2024-02-11

**Authors:** V. Kavimani, Sivarama Krishna Lakkaboyana, Herri Trilaksana, Leonard I. Atanase

**Affiliations:** 1 Department of Chemistry, Prathyusha Engineering College, Chennai 600025, India; kavipathy06@gmail.com; 2 Department of Chemistry, Vel Tech Rangarajan Dr. Sagunthala R&D Institute of Science and Technology, Avadi, Chennai 600062, India; svurams@gmail.com; 3 Department of Physics, Faculty of Science and Technology, Airlangga University, Surabaya 60115, Indonesia; 4 Faculty of Medical Dentistry, Apollonia University of Iasi, 700511 Iasi, Romania; 5 Academy of Romanian Scientists, 050045 Bucharest, Romania

**Keywords:** polyesters, mechanical properties, in vitro degradation, swelling degree, catalyst-free melt polycondensation, elastomer

## Abstract

A new family of polyester-based copolymers—poly(sorbitol adipate-co-ethylene glycol adipate) (PSAEG), poly(sorbitol adipate-co-1,4 butane diol adipate) (PSABD), and poly (sorbitol adipate-co-1,6 hexane diol adipate) (PSAHD)—was obtained with a catalyst-free melt polycondensation procedure using the multifunctional non-toxic monomer sorbitol, adipic acid, and diol, which are acceptable to the human metabolism. Synthesized polyesters were characterized by FTIR and ^1^H NMR spectroscopy. The molecular weight and thermal properties of the polymers were determined by MALDI mass spectroscopy, differential scanning calorimetry (DSC), and thermogravimetric analysis. The degradation rate was investigated, at 37 °C, in 0.1M NaOH (pH 13) and in phosphate-buffered solution (PBS) at pH 7.4. It was found that the polymers degraded faster in NaOH (i.e., in a day) compared to their degradation in PBS, which was much slower (in a week). The highest degradation rate was noticed for the PSAEG sample in both media, whereas PSAHD was the most stable polymer at pH 7.4 and 13. A reduced hydrophilicity of the polymers with diol length was indicated by low swelling percentage and sol content in water and DMSO. Mechanical studies prove that all the polymers are elastomers whose flexibility increases with diol length, shown by the increase in percentage of elongation at break and the decrease in tensile stress and Young’s modulus. These biodegradable copolymers with adaptable physicochemical characteristics might be useful for a broad variety of biological applications by merely varying the length of the diol.

## 1. Introduction

Devices made of plastic are being produced worldwide in an excessive volume [1]. This is because a wide range of functional polymers with various mechanical or chemical characteristics are readily available [2,3,4]. The majority of these materials, unfortunately, cannot be biodegraded. As a result, there is a corresponding rise in plastic trash generation, which adds to environmental degradation [5,6]. Synthetic biodegradable polymers provide greater flexibility than metals and ceramics and may be tailored to improve tissue responsiveness, biodegradability, biocompatibility, and physical qualities. Utilizing degradable plastics, ideally made from renewable resources, is the obvious the first step in addressing this problem. The class of polyesters with (bio)degradable qualities now dominates various application domains [7,8,9]. Different types of enzymes, such as esterase, cutinase, and lipase, are known as being highly active for the degradation of polyester-based polymers [7].

These biomaterials, which are natural-based polymers that may be degraded, are more frequently utilized as scaffolds for tissue engineering, as nanocarriers for medicinal purposes, and as cosmetics [10,11].

Aliphatic polyesters play a predominant role as biodegradable polymers as they contain potentially hydrolysable ester bonds and relatively short aliphatic chains in their macromolecules, and are the most representative examples of environmentally relevant polymeric materials [5,6,12,13]. On the other hand, they often lack good mechanical and physical properties, including unbending and bulk deterioration, which can be compensated by the development of copolymer structures of diols and diacids of various lengths that are both versatile and show high-performance in mechanical applications [14,15,16].

To modify or enhance the physical and chemical properties of polymeric materials, the copolymerization process is a powerful tool. Monomers are selected according to the following set of criteria: (a) non-hazardous, cheap, and easy to obtain from renewable sources; (b) wide application to make any kind of cross-linked network; (c) it is easy to obtain, by polycondensation, which are biodegradable; and (d) biocompatible [17]. A large family of polyols is made up of sugar alcohols with chains of three, four, or six carbon and hydroxyl groups, like glycerol, erythritol, mannitol, malitol, and sorbitol. Polyol copolymerizations with diacids may produce polymers with more hydroxyl groups along the backbone and in the branches. As polyol building blocks, diacids have been looked at as a way to make polyol-based polyesters. The ratio of each initial monomer, reaction time, temperature, and pressure, in addition to other variables, can influence the properties of the resulting polymers [18]. This study selected a mixture of adipic acid and diols with sorbitol as its monomers. Utilizing natural raw materials also has the benefit of being upgradable (renewable) in order to maintain high sustainability and the capacity to maintain a presence [19,20]. The polymer had a polar functional group added to it in order to increase the material’s biodegradability and biocompatibility. In contrast to several other alditols like erythritol (121 °C) and mannitol (165 °C), sorbitol has a lower melting point (95 °C). As sorbitol enters a liquid phase at relatively low temperatures, this makes sorbitol copolymerizations with diacids easier. Like other sugar alditols, it has a large number of hydroxyl groups that make stronger hydrogen bonds between chains and serve as reactive sites for functionalization with other groups. Various sorbitol-based polyesters have been synthesized, like poly(sorbitol sebacate malate) [21], poly(sorbitol citric sebacate), poly(sorbitol tartarate) [20], poly(sorbitol sebacate glutamate) [22], poly(sorbitol adipate-ethylene glycol) [23], and poly(sorbitol sebacate-co-butylene sebacate) [24]. In all the above synthetic sorbitol-based polymers, sorbitol reacted with one or two diacids, but in our study, sorbitol reacted with both a diacid and a diol. So, that diol’s impact on the mechanical, deteriorating, and swelling properties of the polymers was studied with different techniques, such as FTIR and ^1^H NMR spectroscopy, MALDI mass spectroscopy, differential scanning calorimetry (DSC), and thermogravimetric analysis. Moreover, the degradation rate was investigated in 0.1M NaOH (pH = 13) and in PBS at pH 7.4 and 37 °C. The benefits of employing sorbitol as a monomer include its ability to be totally decomposed, and having inexhaustible sources and multiple functional gatherings that allow for the formation of networks with arbitrary cross-link densities which are of interest in the biomedical field.

## 2. Materials and Methods

### 2.1. Materials

Merck AR samples of adipic acid were used. Lancaster samples of high-purity sorbitol, Ethylene glycol,1,4-Butane diol and 1,6-Hexane diol were used. Methanol (MERCK, Darmstadt, Germany) was refluxed over quicklime for six hours and distilled (b.pt. 65 °C). Ethanol (RANCHEM, Delhi, India) was purified by a similar method (b.pt.78 °C). AnalaR samples of dimethyl sulphoxide, dimethyl formamide, tetrahydrofuran, and 1, 4 dioxane were used. 

### 2.2. Synthesis of Copolymer Samples

A series of copolyester samples—PSAEG, PSABD, and PSAHD—were synthesized with a catalyst-free melt polycondensation method. In the synthesis of each polyester, sorbitol, adipic acid, and diol are used. The synthesis of copolyester poly(sorbital adipate-co-ethylene glycol adipate) (PSAEG) is described as follows. Equimolar amounts of sorbitol, adipic acid, and ethylene glycol [S:AA:EG = 1:1:1] were taken in a three-necked round-bottom flask and the monomer mixture was first heated up to 150–160 °C, followed by mixing at 140–145 °C for 1 h under a constant stream of nitrogen. The pre-polymer thus obtained was dissolved in 1,4-dioxane (20% *w*/*w* solution) and the resulting pre-polymer solution was used for film preparation without further purification. Films for structural analyses were prepared by casting into a Teflon Petri dish; they were then left in an oven with a slowly increasing temperature from 80 °C to 150 °C for 2–3 days for solvent evaporation and further polyesterification (curing) of the pre-polymers.

**Figure 1:**
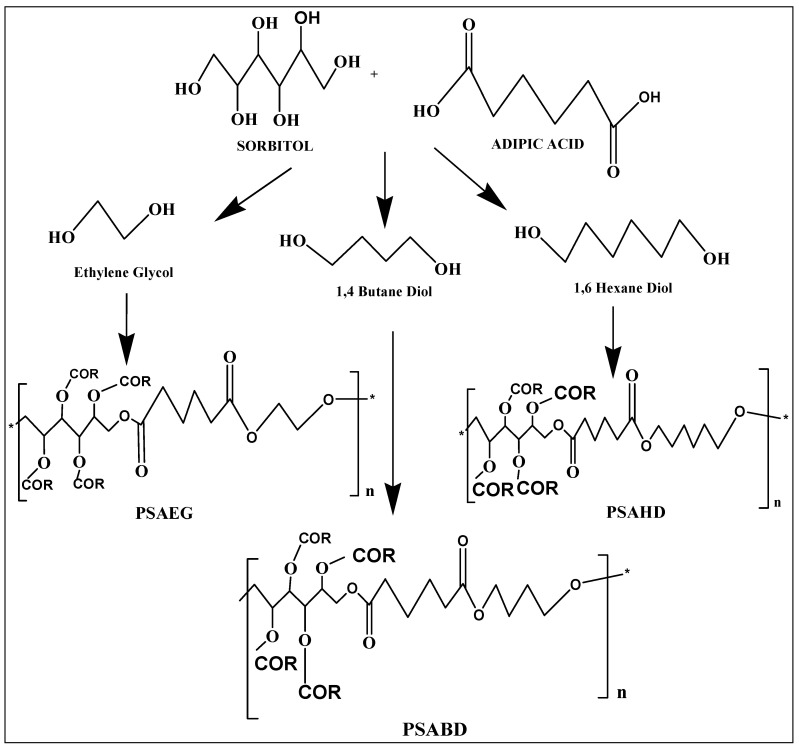
Scheme of poly(sorbitol adipate-co-diol adipate).

The same method was used to synthesize poly(sorbitol adipate-co-1,4 butanediol adipate) (PSABD) and poly(sorbitol adipate-co-1,6 hexanedioladipate) (PSAHD). In this synthesis process, three distinct copolymers (PSAEG, PSABD, and PSAHD) were obtained using three different diols (ethylene glycol, 1,4-butanediol, and 1,6-hexanediol) to examine how mechanical and physical characteristics are affected by diols. After drying, the copolymers were kept for later use in desiccators. The resulting polymers were cross-linked at the reaction groups shown in Figure 1. The –OR in Figure 1 indicates both cross-linked groups and/or –OH groups [20,25]. In Figure 1, the scheme of the synthesis of the copolymers is shown.

### 2.3. Characterization

#### 2.3.1. Solubility Test

Polymer solubility prediction in solvents is one of the most important applications of solubility parameters. The likelihood of the solute’s solubility in the given solvent increases with the proximity of the solubility parameters between the solute and the solvent. When the solvent’s solubility characteristics match those of the polymer, the swell volume, or solvent absorption, will peak in the case of a cross-linked polymer. Solubility tests were performed on copolyester samples using several solvents, including 1,4-dioxane, chloroform, tetrahydrofuran, ethanol dimethyl formamide, water, dimethyl sulfoxide, methanol, and ether.

#### 2.3.2. Analysis by Use of FTIR Spectrometry (Fourier-Transform Infrared)

Solution casting was used to create pre-polymer samples over a KBr crystal using a 5% pre-polymer solution in 1,4-dioxane. A Perkin Elmer IR Spectrometer (PerkinElmer, Shelton, CT, USA) was used to record the IR spectra of each sample between the wavelengths of 700 cm^−1^ and 4500 cm^−1^.

#### 2.3.3. Spectroscopic Analysis Using Nuclear Magnetic Resonance

The three pre-polymers were mixed with 1,4-dioxane, then dissolved in water, settled in water, filtered, and then dried in the air. To acquire the ^1^H and ^13^C NMR spectra of the polymer samples, we used deuterated dimethyl sulfoxide (DMSO-d_6_) as the solvent and tetra methyl silane as the internal reference in a 400 MHz Bruker NMR (Bruker AXS Inc., Madison, WI, USA) spectrometer.

#### 2.3.4. Matrix-Assisted Laser Desorption/Ionization Mass Spectrometry (MALDI-MASS) Analysis

MALDI-MASS analyses were carried out using a Bruker Daltonics instrument. Dihydroxy benzoic acid was used as matrix and NaI was used as the ionizing agent. After optimization, pre-polymers were deposited on a MALDI plate using the layer-by-layer method [26].

#### 2.3.5. Thermal Analysis

Thermal properties of the copolyesters were analyzed on a Perkin-Elmer pyris I Differential Scanning Calorimeter and a thermogram, and were recorded at a scanning rate of 20 °C/min. Differential scanning calorimetry (DSC) thermograms recorded in the range of −70 °C to 500 °C under a nitrogen atmosphere. Similarly, TGA thermograms were observed under the flow of nitrogen gas (50 mL/min) at a scanning speed of 10 °C/min in the range of 50–600 °C. The glass transition temperature, T_g_, was measured to explain the nature of the polymers.

#### 2.3.6. In Vitro Degradation of Polymers

In order to determine the degree of disintegration of disc-shaped samples (7 mm in diameter and 1–1.5 mm thickness), 10 mL of phosphate-buffered saline (pH 7.4) and 0.1 M NaOH (pH = 13) were added to a test tube. Phosphate-buffered saline and NaOH solutions were incubated with the specimens at 37 °C for predetermined periods of time, respectively. The samples had been dried until they reached a constant weight after being incubated, cleaned with distilled water, incubated in ethanol, and afterwards weighed. Equation (1) was utilized to determine each polymer’s mass loss percentage (%*M_loss_*):

(1) $$\%M_{loss}=\left[\frac{M_{0}-M_{d}}{M_{0}}\right]\times 100$$

with the polymer sample’s starting mass (*M*_0_) and final mass (*M_d_*), respectively.

#### 2.3.7. Polymer Sol Concentrations and Swelling Properties in DMSO and Water

A typical procedure used for the determination of a polyester’s swelling degree in DMSO and distilled water is the following: copolymer films are punched out into discs of 10 mm diameter and 1–1.5mm thickness, and are placed at room temperature (27 °C) in 15 mL each of DMSO and distilled water. At regular intervals, the discs are removed from the solvent, and after wiping the surfaces with lint-free paper, their weights are measured. The following expression was used to determine each disc’s percentage swelling (Equation (2)):

(2) $$\%\;\mathrm{Sweeling}\;\mathrm{or}\;\mathrm{Hydration}=\left[\frac{M_{w}-M_{0}}{M_{0}}\right]\times 100$$

where *M*_0_ and *M_w_* indicate, respectively, the disc’s dry and wet mass.

Drying the discs until they all weighed the same after the swelling tests allowed the sol content to be determined using Equation (3):

(3) $$\%\mathrm{Sol}\;\mathrm{content}=\left[\frac{M_{d}-M_{0}}{M_{0}}\right]\times 100$$

where *M*_0_ = disc masses in pre-swelling (dried) states and *M_d_* = disc masses in post-swelling (dried) states.

#### 2.3.8. Mechanical Properties

Using universal testing equipment (S.C. Dey Co., Kolkata, India) fitted with a 500N load cell and data-recording software, the room-temperature, mechanical characteristics of the PSAEG, PSABD, and PSAHD copolymers were determined. A standard test method (ASTM D638) was used in order to measure the samples’ Young’s modulus, tensile strength, and elongation at break with a speed of 50 mm/min. Five samples of polymer thin film, having dumbbells shape and 15 cm × 2 cm × 1 mm (Length × Width × Thickness), were prepared and pulled at a strain rate of 10 mm/min. The initial incline in the tensile stress vs. strain was used to derive the Young’s modulus. The cross-link density and molecular weight between cross-links were calculated using Equation (4), which was derived from the theory of rubber elasticity [24].

(4) $$n=\frac{E_{0}}{3RT}=\frac{\rho }{M_{c}}$$

where *n* = number of active network chain segments per unit volume (mol/m^3^); *M_c_* = molecular weight between cross-links (g/mol); *T* = absolute temperature (298 K); *E*_0_ = Young’s modulus (Pa); and *R* is the universal gas constant (8.314 J/mol. K).

#### 2.3.9. Scanning Electron Microscope

A HITACHI S-3000 scanning electron microscope was used to examine the polyester film structures and morphologies (HRSEM). To ensure the films were well conducted by the electron beam, gold was sputtered over them. The accelerating voltage was 10,000 V, the probe current was 45 A, and the counting time was 60 s.

## 3. Results and Discussion

### 3.1. Solubility Studies

Table 1 shows the solubility data for all the synthesized copolyesters. It appears that the samples were completely insoluble in water, ethanol, and methanol, partially soluble in acetone, and completely soluble in 1,4 dioxane, chloroform, and DMSO. As the synthesized polyester completely dissolves in 1,4 dioxane, the dissolved pre-polymer can be converted to thin films to determine its mechanical property.

### 3.2. Analysis Using Fourier-Transform Infrared (FTIR) Spectrometry

**Figure 2:**
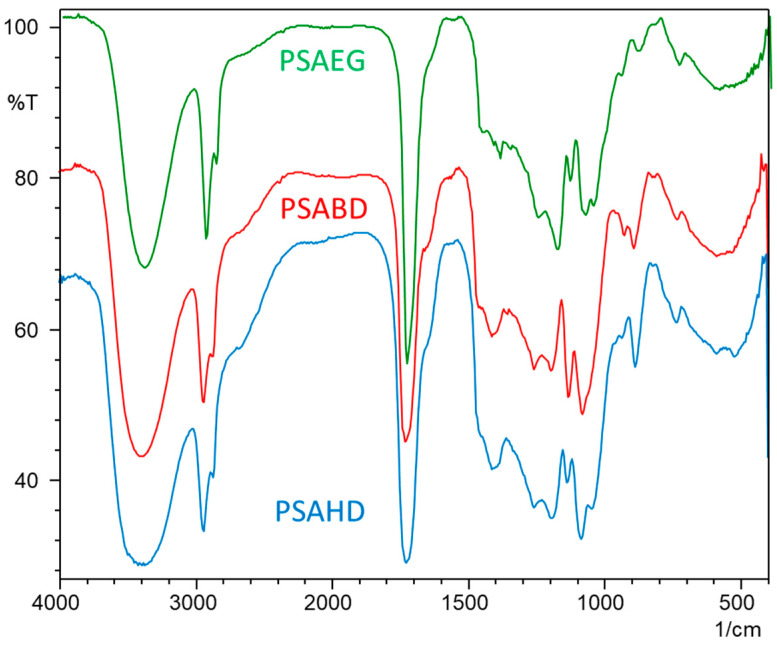
FTIR spectra of copolymers PSAEG, PSABD, and PSAHD.

FTIR spectra of the copolyester poly(sorbitoladipate–co-diol adipate) (PSAEG, PSABD, and PSAHD) samples are provided in Figure 2. Four groups that are typical of polyesters made from sugar alcohols can be found: –C–O–C groups are assigned between 1178 and 1180 cm^−1^; the signal between 1726 and 1729 cm^−1^ is characteristic to C=O groups; the signal between 2931 and 2933 cm^−1^corresponds to CH_2_ groups; and free –OH groups are observed between 3448 and 3450 cm^−1^. Analyzing the spectra of the polymer before and after cross-linking, it can be noticed that the –C–O–C signal intensities increase, whereas the –OH signal intensities decrease. This behavior is due to the ester bonds formed between –OH groups and unreacted molecules of dicarboxylic acids, which are involved in the cross-linking mechanism. The ester carbonyls of PSAEG, PSABD, and PSAHD were all observed at 1726.29 cm^−1^, 1728.21 cm^−1^, and 1726.30 cm^−1^, respectively. The aliphatic C–H of PSAEG, PSABD, and PSAHD bunch balance elongated at 2841.14 cm^−1^, 2843.07 cm^−1^, and 2841.15 cm^−1^, and their corresponding vibration modes were found at 1172.64 cm^−1^, 1174.64 cm^−1^, and 1181.81 cm^−1^ [20,25,27].

### 3.3. Spectroscopic Analysis of Nuclear Magnetic Resonance (NMR)

#### 3.3.1. ^1^H NMR Analysis

**Figure 3:**
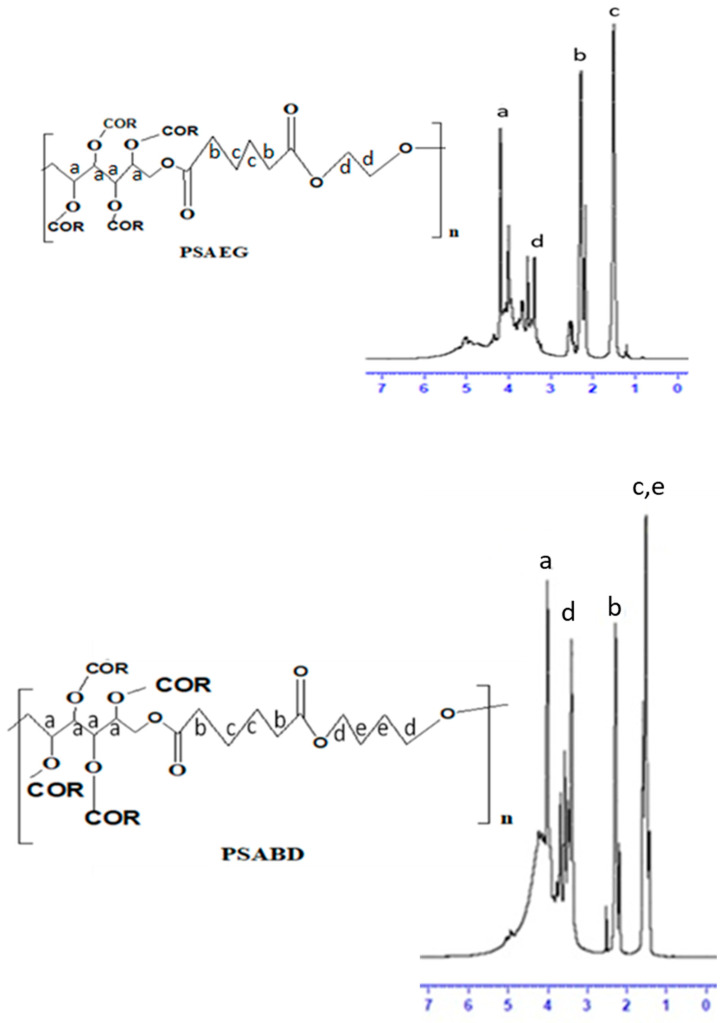

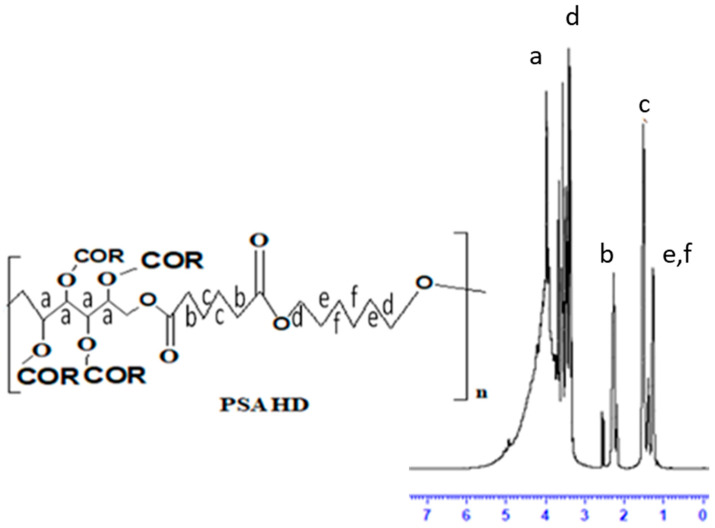
^1^H NMR spectra of PSAEG, PSABD, and PSAHD samples.

The ^1^H NMR spectra of all samples are displayed in Figure 3. The protons n –OCH_2n_ [CH(OH)]nCH_2_O– from sorbitol were ascribed to the peaks between 3.5 and 5.5 ppm [20]. The core methylene (–CH_2_) protons and terminal protons of adipic acid were blamed for the numerous peaks at 2.21 and 1.51 ppm that appeared in all three spectra [28]. A multiple appears in PSAEG at 3.4 ppm due to the terminal methylene protons of ethylene glycol. In PSABD, the peaks that appear at 3.4 and 1.5 are attributed to terminal methylene protons and central methylene protons of 1,4 butane diol [29,30]. In PSAHD, the peaks appearing at 3.4, 1.6, and 1.4 ppm are due to the core methylene and terminal methylene protons of 1,6 hexane diol whose terminal protons appear at 3.6 ppm [29,30].

#### 3.3.2. ^13^C NMR Analysis

**Figure 4:**
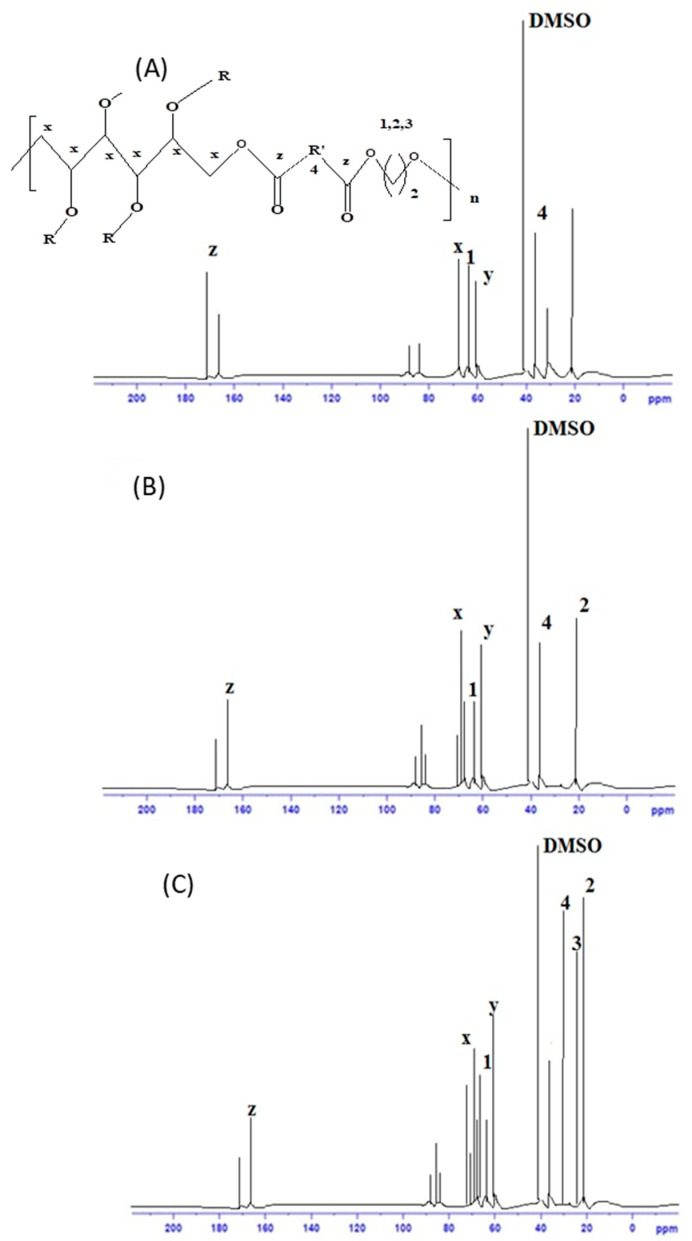
^13^C NMR spectra of PSAEG (**A**), PSABD (**B**), and PSAHD (**C**) samples.

The ^13^C NMR spectra are provided in Figure 4.

The first two resonance peaks for sorbitol in Figure 4 correspond to the terminal carbon and are located at 65.8, 66.1, 72.9, 74.3, and 74.5 ppm, respectively. The peaks between 60 and 65 ppm were caused by carbons that were directly linked to oxygen, either from adipic acid (CO–OCH_2_) or from diols (CH_2_–O–CH_2_) [28]. The peak at 173 ppm was caused by adipic acid’s COO–carbon. The core methylene carbon of adipic acid was responsible for the peaks found in all spectra at 33 ppm. In the PSAEG spectrum, ethylene glycol was identified as the source of the methylene peaks at 63.9 and 61.9 ppm. The methylene carbon of butane diol was attributed to the peaks at 63.9 and 24.1 ppm in the PSABD spectrum, while the peaks at 63.9, 28.9, and 24.1 ppm were all attributed to methylene carbon of hexane diol. Lastly, the solvent DMSO created a peak at around 40 ppm.

#### 3.3.3. Molecular Weight and Structural Composition Analysis by MALDI-MASS Spectroscopy

Matrix-assisted laser desorption–ionization MALDI-MASS spectroscopy was first used to investigate biopolymers. It was used to analyze the distribution of monomer residues over the chains. Even though this technique cannot be used to derive values of M_n_, it gives quantitative information about the amounts of certain observed species. Through this analysis, it is possible to observe the composition of the single chains and to analyse the end group.

**Figure 5:**
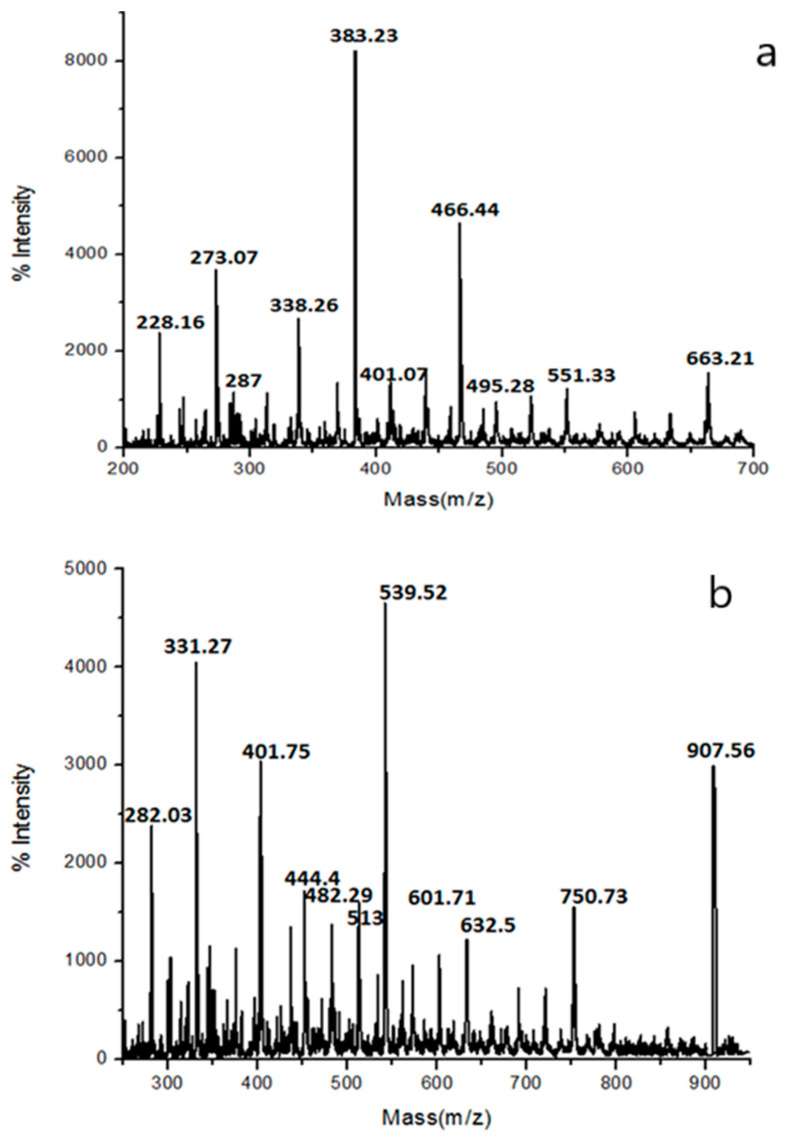
MALDI-MASS spectra of PSAEG (**a**) and PSAHD (**b**) samples.

Furthermore, it was used to acquire evidence for whether sorbitol is incorporated into the polymer chains or not. The variation in the sorbitol/diol/diacid molar ratio of monomers can be used to control the distribution of sorbitol/diacid segments [31]. The molecular weights (Mw) of PSAEG and PSAHD pre-polymers were determined by MALDI-MASS spectroscopy. Figure 5a,b show different spectra corresponding to sorbitol/diols/diacids. From the spectrum, it is observed that the unimodal distribution of ions is characteristic for a step-growth polymerization such as the polyesterification reaction.

The molecular weights of [PSAEG+Na]+ and [PSAHD+Na]+ ions were calculated from the MALDI spectra and the corresponding theoretical and experimental *m*/*z* values are reported in Table 2. These spectra confirmed that the synthesized pre-polymers have low molecular weight. The compositions are shown in Figure 5a,b, and depict that the pre-polymers form linear polyester chains with alternating sorbitol/diacid/diols units, and that they are distributed randomly. Overall, the polymer chains appear to be predominantly hydroxyl-functionalized, even if some carboxylic acid end groups are also present.

### 3.4. Thermal Analysis

Thermal analysis is an analytical technique to investigate the behavior of a sample as a function of temperature. The thermal analysis of polyesters is important because these investigations not only explain the behavior of the polyesters when subjected to high temperature, but also help in establishing criteria for the selection of materials for a specific use. The thermal history of a polymer has a strong influence on the characteristic melting and crystallization temperature.

#### 3.4.1. Thermo gravimetric Analysis

Thermo gravimetric analysis measures the amount and rate of change in mass of a sample as a function of temperature or time in a controlled atmosphere. The measurements are used primarily to determine the thermal and/or oxidative stability of material as well as compositional properties. This technique can analyze materials that exhibit either mass loss or gain due to decomposition, oxidation, or loss of volatiles such as moisture. TGA measurements provide valuable information that can be used to select materials for certain end-user applications, predict product performance, and improve product quality.

**Figure 6:**
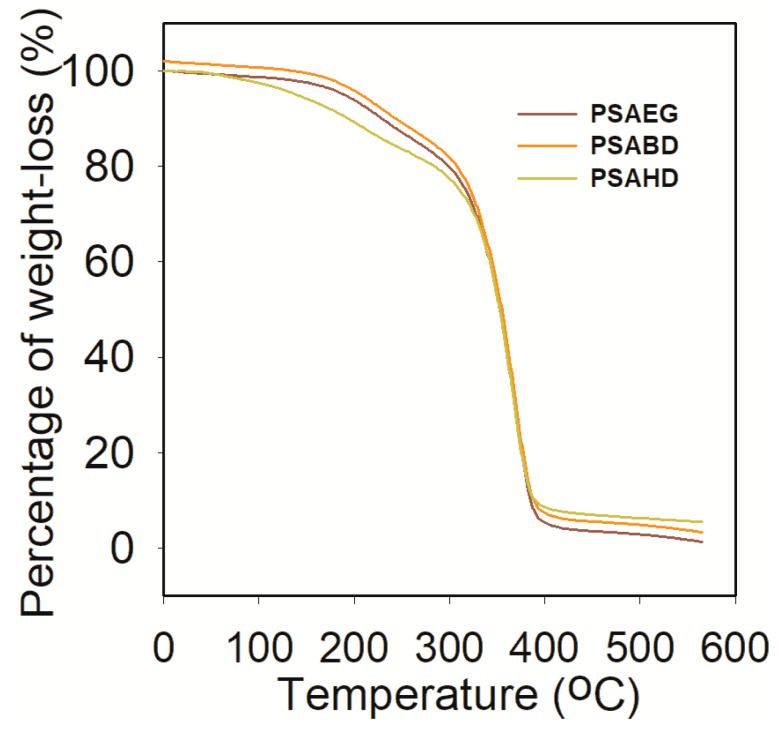
TGA of the PSAEG, PSABD, and PSAHD samples.

The thermal stability of the copolyesters PSAEG, PSABD, and PSAHD is characterized by the onset of temperatures at 25%, 50%, and 75%, and max weight loss, which are referred as T_onset_, T_25%_,T_50%_, T_75%_,and T_max%_, with residual mass at 594.9 °C, respectively, as given in Table 3. The TG thermograms are shown in Figure 6.The decomposition of the polymer starts at 350 °C and reaches nearly 100% at 420 °C. It is a common practice to consider 50% weight loss as an indicator for structural destabilization. From Table 3, it is observed that the thermal stability of the copolyesters decreases with the increase in the number of methylene units in the diol.

#### 3.4.2. Differential Scanning Calorimetry (DSC)

**Figure 7:**
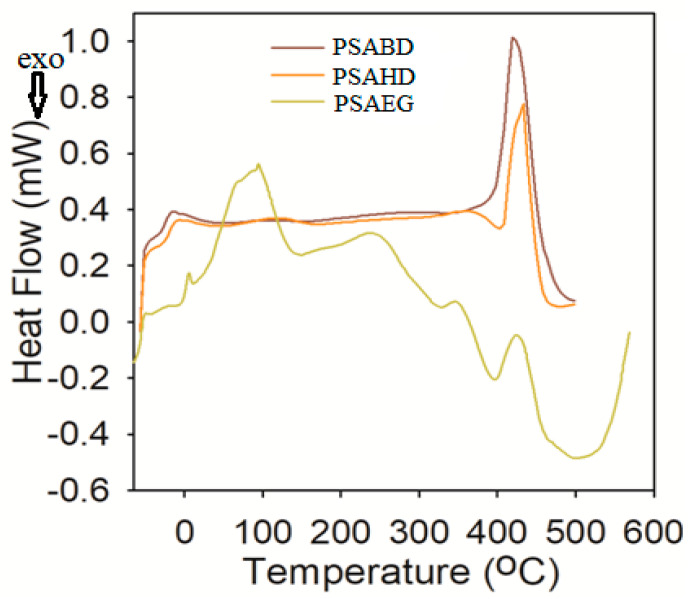
DSC thermograms of the PSAEG, PSABD, and PSAHD samples.

Differential scanning calorimetry (DSC), a physical characterization method, is used to study the thermal behavior of neat polymers, copolymers, polymer blends, and composites. The thermal behavior, that is, the glass transition temperature (T_g_), of the PSAEG, PSABD, and PSAHD copolyesters was obtained from DSC thermograms and the values are given in Table 4. The thermograms obtained from the DSC analyses are presented in Figure 7. As shown in the DSC thermograms, all the synthesized copolyesters have only T_g_ values, showingthat these copolyesters are amorphous in nature. All T_g_ values lie below room temperature, showing that these copolyesters are thermoset elastomers which are suitable for tissue engineering and drug delivery applications [20,30]. The DSC data show that the T_g_ value increases from −59.2 °C to −23.7 °C with increase in diol length, which is due to the increase in chain rigidity associated with the number of hydrogen bonds. Similar results have been obtained for xylitol-based polymers [18,32]. The additional complex peak in the PSAEG curve is due to its decomposition, proving that PSAEG is a less stable polymer when compared with the other two polymers, PSABD and PSAHD, which is already proven in swelling and degradation studies as well.

### 3.5. Swelling Studies of Elastomers in Water and DMSO

**Figure 8:**
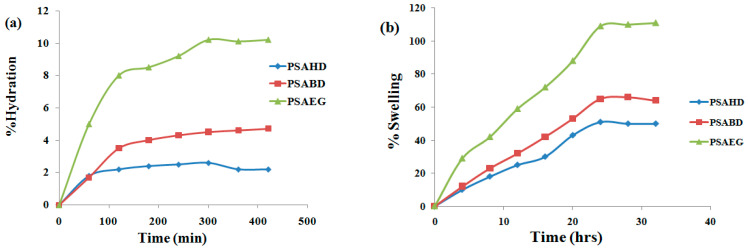
Swelling behavior of the polymers (**a**) water and (**b**) DMSO.

The swelling properties of biomaterials can be crucial to understand their interaction with biological systems. Figure 8 displays the polymer swelling profiles in both water and DMSO. The highest hydration of 10.5% was observed for the PSAEG sample, while the minimum value of 2% was observed for the PSAHD copolymer. All samples achieved their equilibrium state in 2 to 3h. The polymers’ hydratability is affected by the hydrophilicity of their building sequences, as well as the ratio of hydrophilic to hydrophobic segments [33]. Despite the fact that diol and acid are naturally hydrophilic, the synthetic polyester is hydrophobic. Our copolymers demonstrated that the polymer chain length enhances the hydrophobicity. Polymers of ethylene glycol, 1,4-butane diol, and 1,6-hexane diol have increased hydrophobicity and reduced hydration value as a function of diol chain length.

The polymers’ predicted sol contents are displayed in Table 5. The low swelling percentage and sol content show that a cross-linked network with an increased number of ester bonds was successfully obtained and that the unreacted hydrophilic hydroxyl and carboxyl groups are no longer available on the surface of the films [20].

Quite a similar behavior was noticed for the swelling behavior of the samples in DMSO. The cross-linked copolymers are insoluble in DMSO due to their strong intermolecular interactions and hydrogen bonding, whereas the non-cross-linked samples dissolve easily. This was consistent with the FTIR analysis’s finding that hydroxyl and carboxylic groups were present, forming hydrogen bonds [20,34]. As the diol length increases, the polymer becomes non-polar, and there is less swelling in the polar solvent.

### 3.6. In Vitro Degradation of Copolymers

**Figure 9:**
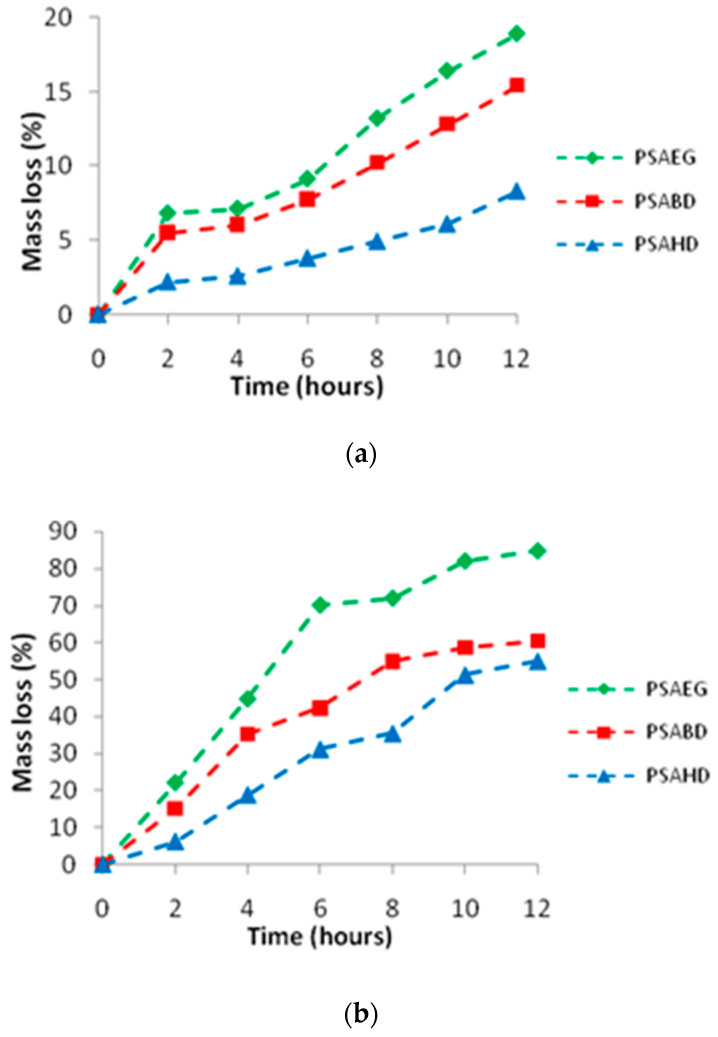
Degradation rate as a function of time (**a**) in PBS (pH 7.4) and (**b**) in 0.1 M NaOH (pH = 13) at 37 °C.

When aliphatic degradable polyesters are exposed to water, hydrolysis causes them to begin breaking down. Under physiological settings, the degradation of all synthetic polyesters was examined in phosphate-buffered solution (pH 7.4) and 0.1 M NaOH (pH 13.0) at 37 °C. In Figure 9, polymer degradation is represented by a gradual decrease in weight as a function of time.

From Figure 9, it can be noticed that the degradation rate at pH 13 is much higher than at pH 7.4. Moreover, in both degradation media, it seems that PSAEG is more easily degradable than the other two copolymer samples. Three factors appear to be in control of how quickly a synthetic polyester degrades: the pH of the fluid in which the degradation occurs, the polymer’s hydrophilicity given by the nature of the monomer, and the cross-link density. By comparing the degradation of copolymers in both media, it appears that polyester degrades quickly in an alkaline environment (i.e., 85% of the degradation is complete in 6 h), whereas only 14% of the same polyester degrades in a PBS solution (pH 7.4). This behavior is in agreement with other studies in the literature [35]. This is because base catalysis induces quicker chain cleavage than polymer swelling. As a result, materials that are soft and hydrophilic will degrade quickly, whereas those that are hard and hydrophobic will withstand hydrolysis longer. The relative rates of degradation of the obtained polyesters showed this logical trend [36]. The hydrophilicity of the polymer rises as the diol chain length decreases, hastening the polymer’s degradation [37]. Accordingly, the synthesized copolyester’s degradation rate decreases in the following order: PSAEG > PSABD > PSAHD.

### 3.7. Mechanical Properties

**Figure 10:**
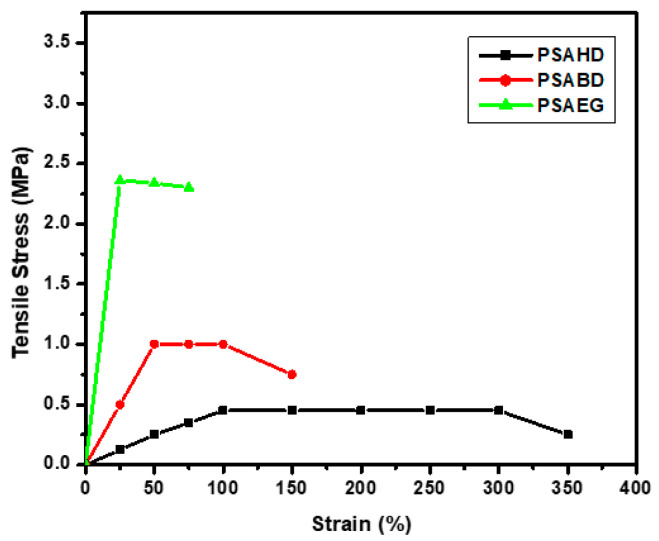
Tensile stress vs. strain curves for PSAEG, PSABD, and PSAHD samples.

The polymers showed properties in tensile testing that were intermediate between those of elastomers. Figure 10 represents the correlation between the tensile stress and strain, and their values in a mechanical study are given in Table 6.

From Table 6, it appears that by increasing the diol chain length, the values of the Young’s modulus, tensile stress, and cross-link density decrease, and elongation at break increases, which shows that the PSAHD is a soft and flexible polymer. These results are in concordance with other studies in the literature [21,32,36,37,38]. Moreover, the obtained results confirm that by increasing the length of the diol, the polymers became softer and more extensible. Furthermore, the data from Table 6 show that it is possible to modulate a wide range of mechanical properties of the copolymers just by varying the diol’s chain length.

### 3.8. Scanning Electron Microscopy

**Figure 11:**
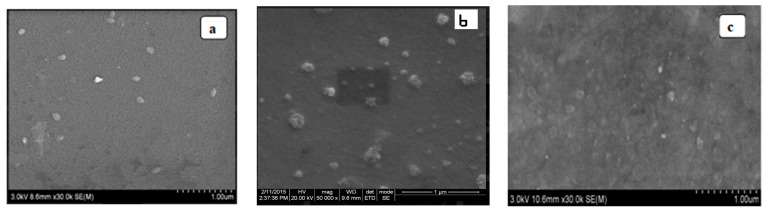
SEM images of (**a**) PSAEG, (**b**) PSABD, and (**c**) PSAHD.

Scanning electron microscopy (SEM) is a highly helpful imaging method that makes use of an electron beam to capture pictures of objects at high resolution. The transitional zone of the copolyester films is seen in Figure 11.

A filtering electron magnifier was used to examine the surface morphology and it was noticed that the surface was smooth with few agglomerations and an accumulation of holes inside the films.

## 4. Conclusions

The polycondensation of sorbitol with adipic acid and diols like ethylene glycol, 1,4-butane diol, and 1,6-hexane diol yielded poly(sorbitol adipate-co-dioladipate) (PSAEG, PSABD, and PSAHD) polyester copolymers. MALDI mass spectra confirmed that the synthesized pre-polymers have low molecular weight. Linear and hydrophilic polyol polyesters are interesting candidates for the next generation of bio-based materials because of their cheap catalyst system and straightforward synthesis technique. Tensile strength and Young’s modulus were both significantly improved after adipic acid was used as a third monomer. From the thermal studies, it is observed that the thermal stability of these copolyesters increases with increase in the number of methylene units in the polymer chain. T_g_ values lies below room temperature, showing that these copolyesters are thermoset elastomers which are suitable for tissue engineering and drug delivery applications.

The research showed that the PSAEG polyester has higher mechanical characteristics compared to PSABD and PSAHD. The degradation rate of the PSAHD copolymer is low due to a higher cross-link density. The three copolymers are all hydrophilic by nature, which is a crucial quality for controlled release and other medicinal uses. These polyesters have many uses in the human body, from ligaments and blood vessels to neurons and tendons, and their physical and mechanical qualities can be tailored by adjusting the curing conditions. In physiological settings, the polymers show a gradual pattern of degradation that may be controlled by changing curing conditions after polymerization. It is proposed that future syntheses will be carried out with different sorbitol-to-polymer ratios in order to obtain a material that is better suited to the conditions found inside the human body and can be used more effectively in biomedical applications.

## Figures and Tables

**Table 1:** Solubility data.

Sample	DMSO	1,4 Dioxane	Acetone	CHCl_3_	Ethanol	Methanol	Water
PSAEG	+++	+++	++	+++	−−−	−−−	−−−
PSABD	+++	+++	++	+++	−−−	−−−	−−−
PSAHD	+++	+++	++	+++	−−−	−−−	−−−

−−−Insoluble; ++ partially soluble; +++ freely soluble.

**Table 2:** MALDI-MASS data of copolyesters.

Samples	Composition	*m*/*z*	
		Experimental	Theoretical
PSAEG	P(A_1_EG_1_)-2Na	226.16	228.16
	P(A_1_EG_1_)-4Na	272.16	273.07
	P(A_1_EG_2_)-2Na	288.23	287.00
	P(A_1_EG_2_)-4Na	334.23	338.26
	P(S_1_A_1_EG_1_)-Na	385.3	383.23
	P(A_2_EG_2_)-2Na	406.32	401.07
	P(S_1_A_1_EG_2_)-2Na	470.37	466.44
PSAHD	P(A_1_HD_1_)-2Na	282.03	282.29
	P(A_1_HD_1_)-4Na	331.27	328.29
	P(S_1_A_1_HD_1_)-Na	444.4	441.46
	P(S_1_A_1_HD_1_)-4Na	513.39	510.46
	P(S_1_A_2_HD)-4Na	632.5	628.55
	P(S_1_A_2_HD_2_)-4Na	750.73	746.75
	P(S_2_A_2_HD_2_)-Na	907.56	911.04

**Table 3:** Thermal decomposition data of copolyesters.

Sample	T_d_ under Nitrogen Atmosphere (°C)					Residual Sample at 594.9 °C (%)
	T_onset_ (°C)	T_25%_ (°C)	T_50%_ (°C)	T_75%_ (°C)	T_max_ (°C)	
PSAEG	34.8	310.7	381.3	406.1	402.5	0.97
PSABD	34.8	313.8	382.7	406.1	403.4	2.74
PSAHD	34.8	314.7	382.0	406.1	399.4	5.21

**Table 4:** Thermal data of copolyesters.

Sample	T_g_ (°C)
PSAEG	−59.2
PSABD	−28.4
PSAHD	−23.7

**Table 5:** Sol content of the copolymer samples.

Sample	Sol Content (%)	
	Water	DMSO
PSAEG	12.0	11.2
PSABD	10.9	5.8
PSAHD	6.8	2.51

**Table 6:** Young’s Modulus, tensile strength, and elongation at break values.

Sample	Young’s Modulus (MPa)	Tensile Stress (MPa)	Cross-Link Density n × 10^3^ (mol/m^3^)	Elongation at Break (%)
PSAEG	3.44 ± 0.03	2.36 ± 0.02	22.23 ± 0.03	62 ± 2
PSABD	0.25 ± 0.01	1.05 ± 0.02	13.1 ± 0.02	142 ± 3
PSAHD	0.09 ± 0.01	0.46 ± 0.01	11.4 ± 0.02	308 ± 5

## Data Availability

The data presented in this study are available on request from the corresponding author.
